# Supplementary material for: In Silico and In Vivo Evaluation of Synthesized SCP-2 Inhibiting Compounds on Life Table Parameters of Helicoverpa armigera (Hübner)
Source: Insects. 2022 Dec 16;13(12):1169. doi: 10.3390/insects13121169 (PMC9782802; doi:10.3390/insects13121169)
Supplement: Supplementary file 1 [file insects-13-01169-s001.zip › insects-2010754-supplementary.pdf]

A) SCP-2-OA-01

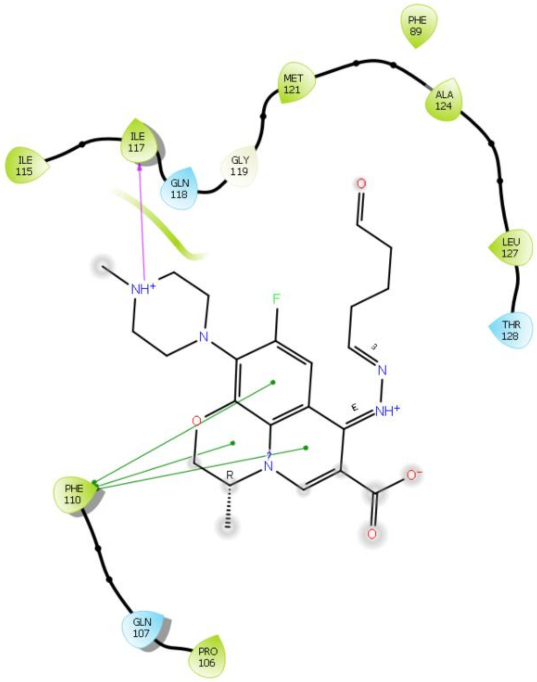

B) SCP-2-OA-02

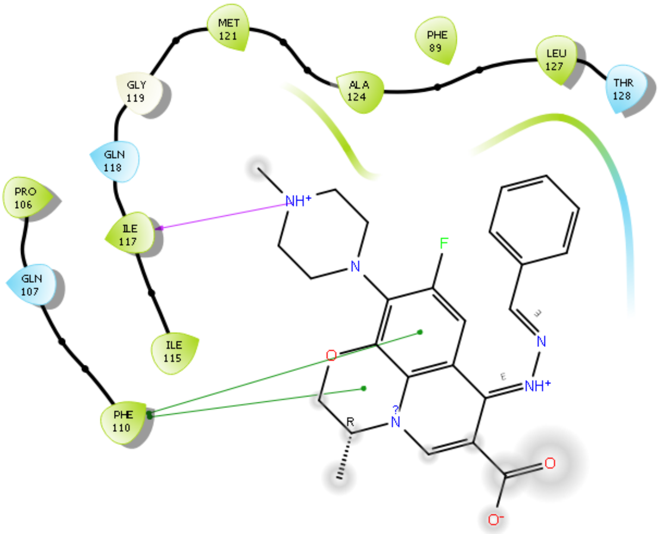

C) SCP-2-OA-03

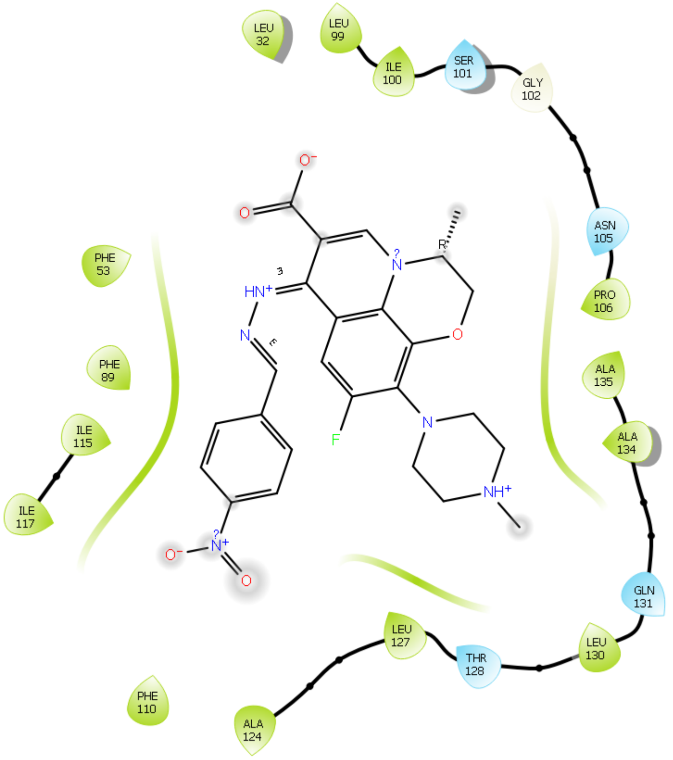

D) SCP-2-OA-04

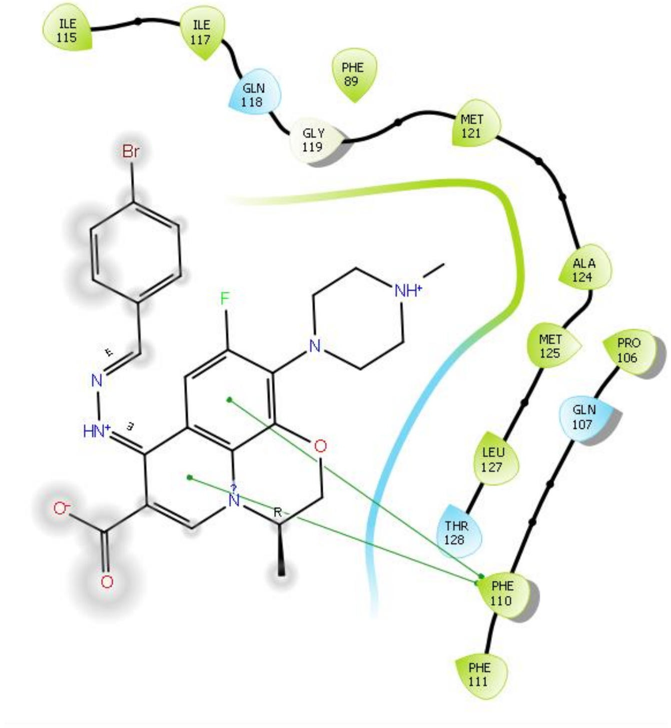

E) SCP-2-OA-05

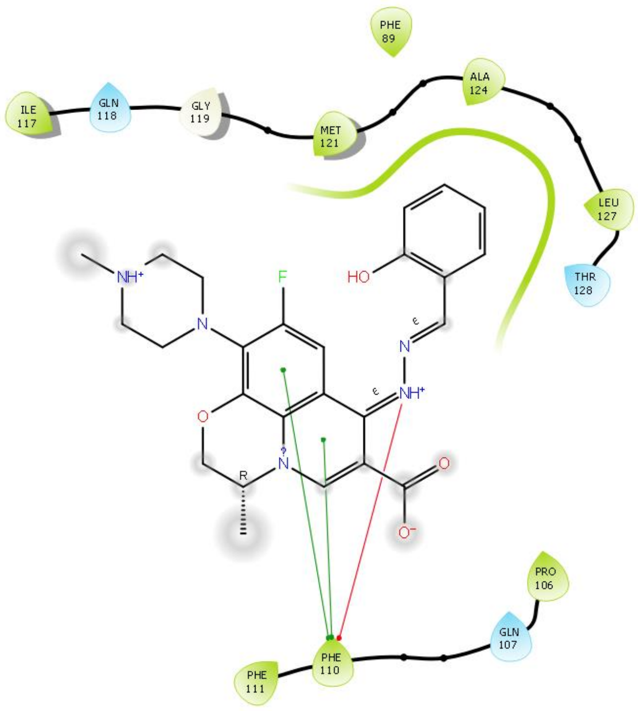

F) SCP-2-OA-06

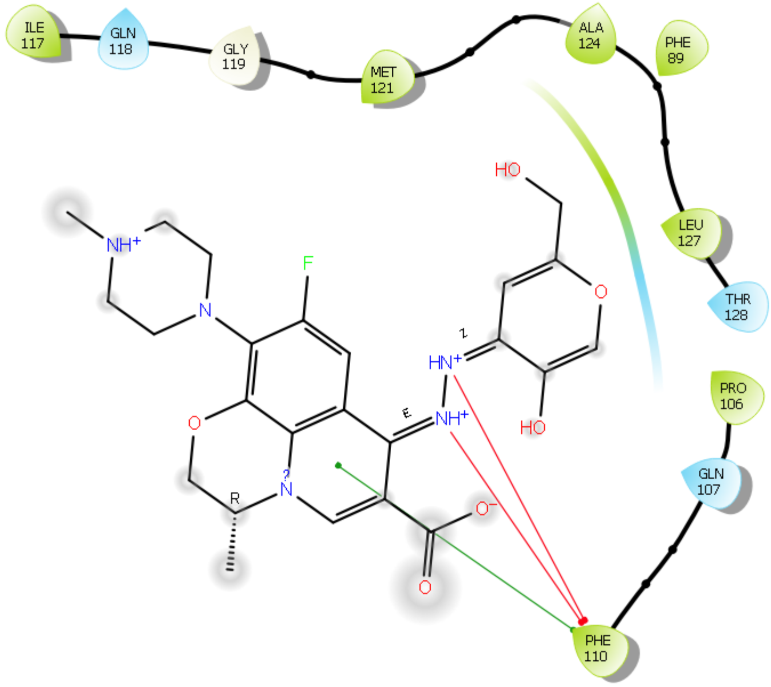

G) SCP-2-OA-07

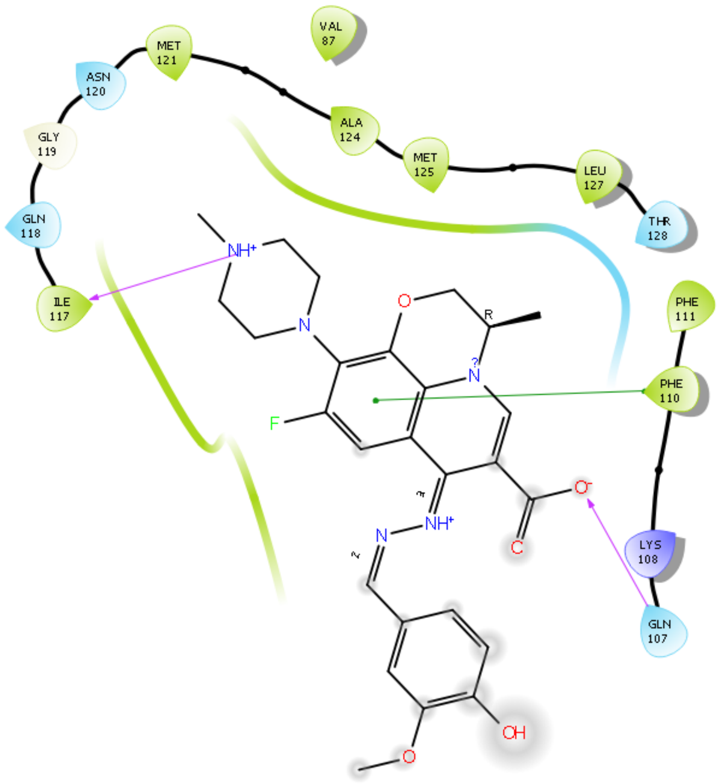

H) SCP-2-OA-08

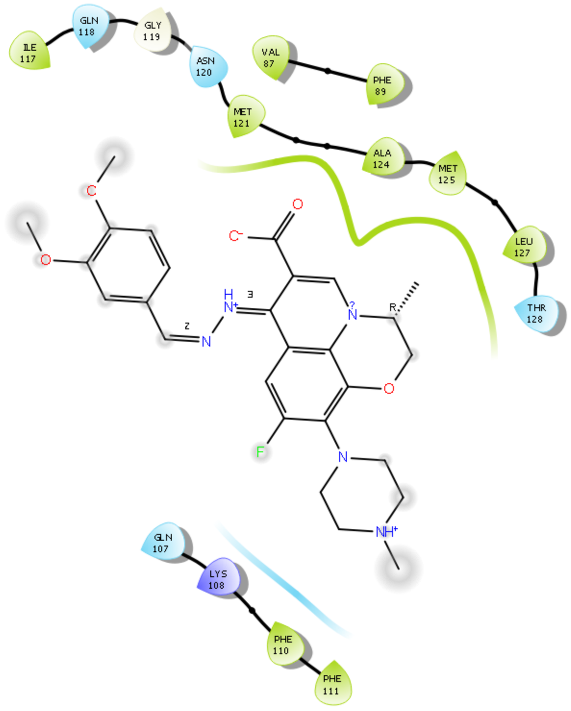

I) SCP-2-OA-09

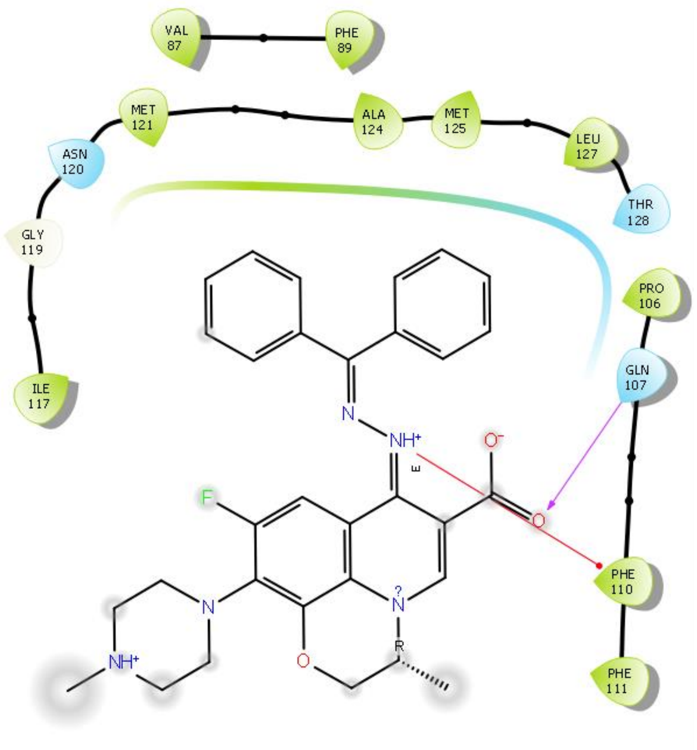

Figure S1: Interactions of the OA series compounds with the SCP2 protein

Table S1: Binding free energy calculations of complexes by MM/GBSA

**BINDING ENERGY COMPONENTS**	**SCP2-OA-02**	**SCP2-OA-06**	**SCP2-OA-09**
**$\Delta G_{bind} = (G_{complex}) - (G_{protein}) - (G_{ligand})$**			
VDWAALS	-34.80 ± 0.45	-37.48 ± 0.54	-38.02 ± 0.42
EEL	-2.78 ± 1.41	2.62 ± 0.85	-6.39 ± 1.18
EGB	17.68 ± 1.35	18.36 ± 0.87	21.19 ± 1.12
ESURF	-4.51 ± 0.04	-4.86 ± 0.05	-4.96 ± 0.04
DELTA G gas	-37.59 ± 1.39	-34.85 ± 1.07	-44.41 ± 1.22
DELTA G solv	13.17 ± 1.35	13.50 ± 0.84	16.22 ± 1.10
DELTA TOTAL	-24.42 ± 0.33	-21.35 ± 0.45	-28.19 ± 0.40

Table S2: Contribution of active site residues in the binding free energies

RESIDUES	SCP2-OA-02	SCP2-OA-06	SCP2-OA-09
VAL87	0.054 ± 0.008	0.08 ± 0.01	-0.171 ± 0.008
PHE89	-0.472 ± 0.026	-0.605 ± 0.047	-0.184 ± 0.015
PRO106	-0.925 ± 0.05	-1.238 ± 0.048	-0.35 ± 0.027
GLN107	-3.544 ± 0.252	-2.16 ± 0.091	-4.008 ± 0.208
PHE110	-2.214 ± 0.137	-4.107 ± 0.073	-3.59 ± 0.065
PHE111	-0.421 ± 0.057	-0.127 ± 0.01	-1.688 ± 0.056
ILE117	-1.779 ± 0.063	-1.502 ± 0.078	-1.921 ± 0.056
GLY119	-0.89 ± 0.137	-0.439 ± 0.077	0.018 ± 0.024
ASN120	0.368 ± 0.043	0.492 ± 0.055	-0.245 ± 0.014
MET121	-2.985 ± 0.086	-3.134 ± 0.084	-2.639 ± 0.077
ALA124	-0.451 ± 0.024	-0.544 ± 0.041	-0.988 ± 0.04
MET125	-1.195 ± 0.055	-0.865 ± 0.051	-0.724 ± 0.038
LEU127	-0.012 ± 0.007	-0.036 ± 0.01	-0.072 ± 0.008
THR128	-0.199 ± 0.027	-0.296 ± 0.022	-0.02 ± 0.048
